# Comment on: Combination of immunosuppressive therapy and nintedanib improves capillaroscopic changes in systemic sclerosis–interstitial lung disease: a case report

**DOI:** 10.1093/rap/rkac024

**Published:** 2022-03-31

**Authors:** Tomohiro Sugimoto, Yusuke Yoshida, Sho Mokuda, Shintaro Hirata

**Affiliations:** Department of Clinical Immunology and Rheumatology, Hiroshima University Hospital, Kasumi, Hiroshima, Japan

Key message

- Immunosuppressants repair nailfold capillary abnormalities in patients with early-stage SSc.

Dear Editor, We read the paper by Matsuda *et al.* [1] with great interest. We believe that the fact that the abnormalities of the nailfold capillaries in patients with SSc were improved by treatment is very important.

As the authors pointed out, few reports exist on the improvements in nailfold capillary abnormalities after immunosuppressive treatment in patients with SSc. Among them, CYC improved the nailfold capillary abnormalities of SSc when using iloprost in five of eight patients [2]. Autologous stem cell transplantation and CYC treatment were compared for the improvement of nailfold capillary abnormalities in patients with SSc, and transplantation alone resulted in improvement in capillaries [3]. The difference between these reports lies in whether the nailfold capillary abnormality was an early lesion. Therefore, we believe that immunosuppressive treatment should improve the abnormalities of nailfold capillaries in the early and active patterns. In a previous report, other drugs were used in combination, and there is not enough evidence on whether monotherapy immunosuppressive agents improve the abnormalities of the nailfold capillaries.

We reported that in five patients with SSc treated with CYC i.v. therapy, findings of enlarged capillaries, giant capillaries and haemorrhage among the nailfold capillary abnormalities improved after 6 months [4]. Additionally, we presented the case of a patient with anti-Scl-70 antibody-positive SSc treated with tacrolimus alone. She also had arthritis, which was shown to be rheumatoid because she tested positive for RF and anti-CCP antibody. The patient was also diagnosed with interstitial pneumonia using CT and was treated with tacrolimus for arthritis and interstitial pneumonia. She underwent a nailfold video-capillaroscopic examination at the first visit and was found to have an abnormal and active pattern according to the criteria of Cutolo *et al.* [5]. She was re-examined after treatment, and her vascular findings had improved (Fig. 1). Combining previous reports with our cases, we believe that immunosuppressive treatment improves vascular lesions in patients with SSc who have early or active patterns of nailfold capillary abnormalities.

**Fig. 1:**
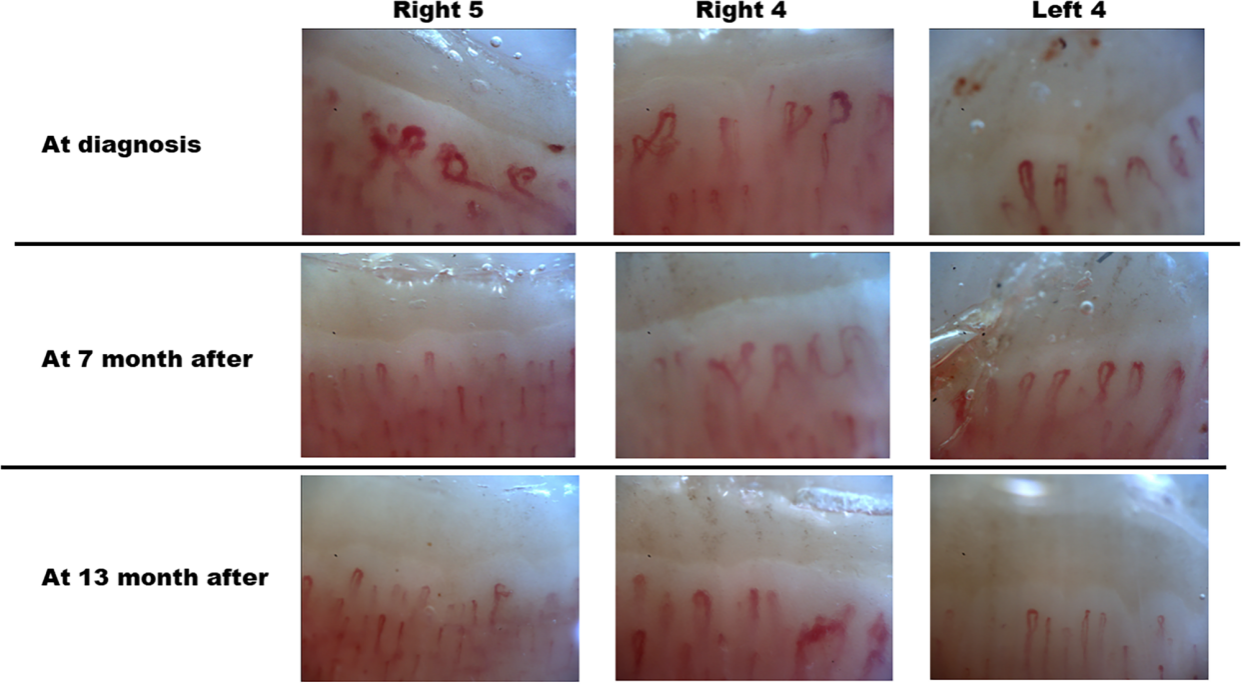
Results of nailfold-video capillaroscopic examination Nailfold-video capillaroscopy can evaluate blood vessels at a magnification of ×200. Images at the time of diagnosis show enlarged capillaries, giant capillaries and haemorrhage. In the images after 7 and 13 months, the haemorrhage has disappeared and the enlarged capillaries and giant capillaries decreased.

In the recent study by Matsuda *et al.* [1], tacrolimus improved the nailfold capillary abnormalities. In addition, the photographs they presented cannot be interpreted unequivocally, because we have confirmed only some of the results; however, we consider them to be early rather than active patterns according to the criteria of Cutolo *et al.* [5]. We consider that the vascular abnormalities in the study by Matsuda *et al.* [1] were mild and could be improved to almost normal. We have previously reported that even in patients with SLE and DM, nailfold capillary abnormalities can be improved by drug therapy without the use of antifibrotic drugs [5, 6, 7]. We believe that further verification is needed to determine whether nintedanib improves nailfold capillary abnormalities. To verify this, it is necessary to determine the effect of nintedanib alone in patients with early-stage SSc without immunosuppressive treatment. We believe that the overall effect of the combination therapy can be understood only after understanding the healing effect of each single agent.

In summary, we argue that early-stage nailfold capillary abnormalities can be improved with immunosuppressive agents alone. As pointed out by Matsuda *et al.* [1], further verification is needed regarding the ability of nintedanib to repair nailfold capillary abnormalities.

*Funding:* This work was supported in part by Japan Society for the promotion of science KAKENHI (grant numbers 19K18499 to S.M. and 19K07940 to S.H.), Mitsubishi Foundation to S.M., Takeda Science Foundation to S.M., Mochida Memorial Foundation for Medical and Pharmaceutical Research to S.M., Japanese Respiratory Foundation Grant to S.M. and Japan Rheumatism Foundation to S.M.

*Disclosure statement:* The authors have declared no conflicts of interest.

*Patient consent:* Written consent was obtained from the patient regarding the publication of this case. This study was approved by the clinical ethics committee of Hiroshima University Hospital (approval number: E-1393; approval date: 18/Oct/2018).

## Data availability statement

The data underlying this article will be shared on reasonable request to the corresponding author.
